# Asymptomatic-anaplasmosis confirmation using genetic and serological tests and possible coinfection with spotted fever group *Rickettsia*: a case report

**DOI:** 10.1186/s12879-020-05170-9

**Published:** 2020-06-30

**Authors:** Jiyeon Yoo, Jong-Hoon Chung, Choon-Mee Kim, Na Ra Yun, Dong-Min Kim

**Affiliations:** 1 College of Medicine, Chosun University, Gwangju, South Korea; 2 Departments of Internal Medicine, College of Medicine, Chosun University, 588 Seosuk-dong, Dong-gu, Gwangju, 61453 Republic of Korea; 3 Premedical Science, College of Medicine, Chosun University, Gwangju, Republic of Korea

**Keywords:** Anaplasmosis, *Anaplasma phagocytophilum*, Spotted fever group rickettsiosis, Tick bites

## Abstract

**Background:**

Anaplasmosis is an emerging acute febrile disease that is caused by a bite of an *Anaplasma phagocytophilum*–infected hard tick. As for healthy patients, reports on asymptomatic anaplasmosis resulting from such tick bites are rare.

**Case presentation:**

A 55-year-old female patient visited the hospital with a tick bite in the right infraclavicular region. The tick was suspected to have been on the patient for more than 10 days. PCR and an indirect immunofluorescence assay (IFA) were performed to identify tick-borne infectious diseases. The blood sample collected at admission yielded a positive result in nested PCR targeting *Ehrlichia*- or *Anaplasma*-specific genes *groEL* and *ankA*. Subsequent sequencing confirmed the presence of *A*. *phagocytophilum*, and seroconversion was confirmed by the IFA involving an *A*. *phagocytophilum* antigen slide. PCR detected no *Rickettsia*-specific genes [outer membrane protein A (*ompA*) or surface cell antigen 1 (*sca1*)], but seroconversion of spotted fever group (SFG) rickettsiosis was confirmed by an IFA.

**Conclusions:**

This study genetically and serologically confirmed an asymptomatic *A*. *phagocytophilum* infection. Although SFG rickettsiosis was not detected genetically, it was detected serologically. These findings indicate the possibility of an asymptomatic coinfection: anaplasmosis plus SFG rickettsiosis. It is, therefore, crucial for clinicians to be aware of potential asymptomatic anaplasmosis following a tick bite.

## Background

Anaplasmosis is an infection that is caused by a bite of an *Anaplasma phagocytophilum*–infected hard tick. It is an acute febrile disease that is characterized by a high fever after a latency period of 7–10 days [1]. The clinical manifestations of this disease can include an array of nonspecific symptoms including a fever, chills, headache, and muscle ache as well as possible additional symptoms such as vertigo, upper gastrointestinal tract bleeding, and seizures [2].

Anaplasmosis is an infectious disease that has become increasingly prevalent in Korea since the first reported case in 2014 [3]. Spotted fever group (SFG) rickettsiosis features signs and symptoms such as a high fever, flu-like symptoms, eschar around the bite, papules, and rashes. It may also affect other organs such as the nervous system [4].

There have been cases of asymptomatic anaplasmosis in animals that have required genetic diagnosis (camels and horses) [5, 6] or genetic and serological diagnosis (dogs and sheep) [7, 8]. Nevertheless, human cases of asymptomatic anaplasmosis are rare [9].

In this study, we tested for asymptomatic anaplasmosis after a tick bite and a suspected coinfection with SFG *Rickettsia* in an otherwise healthy patient.

## Case presentation

### Case

**Fig. 1:**
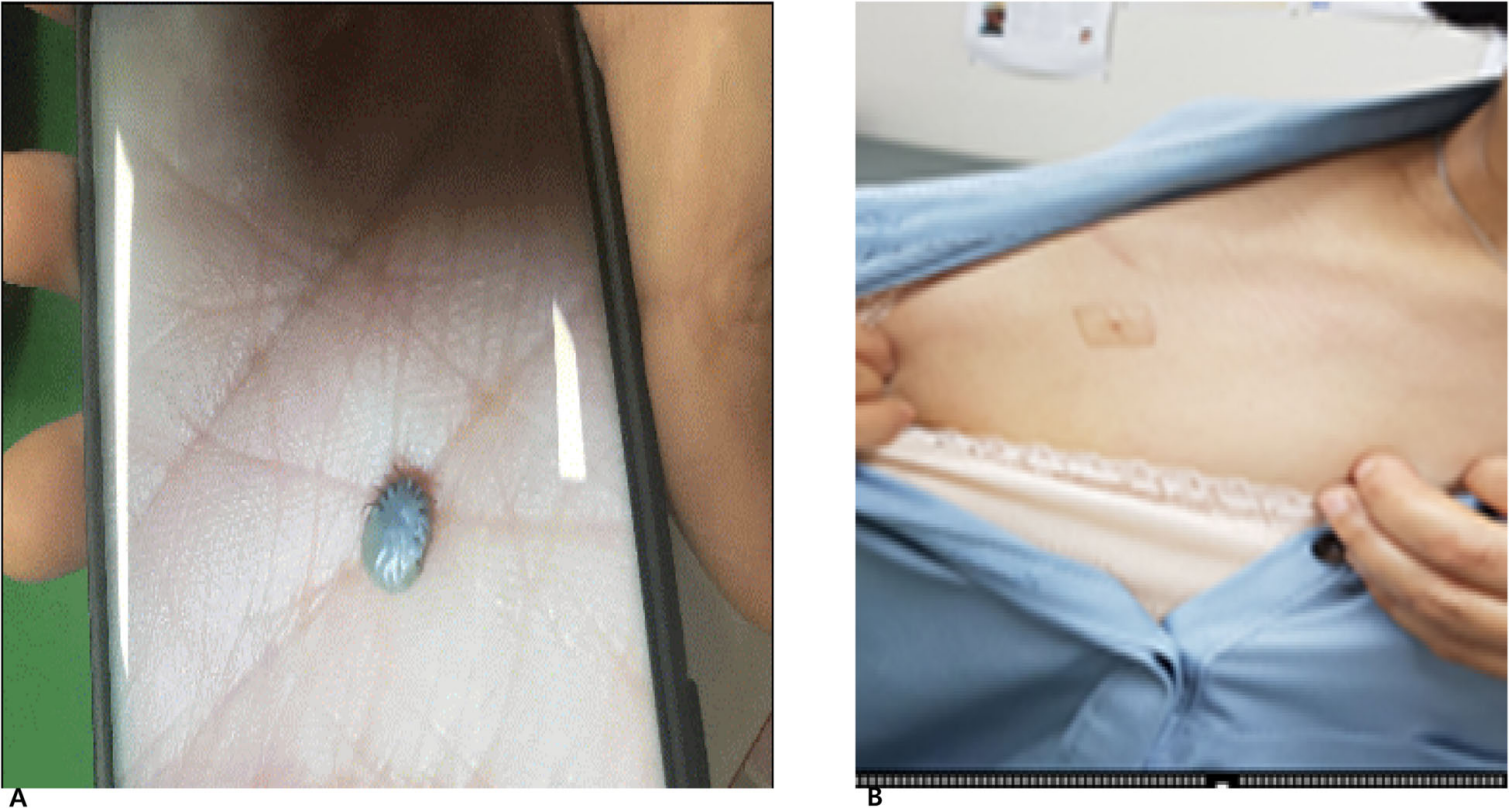
The tick image captured using the patient’s cell phone (**a**). The tick bite lesion located under the right clavicle (**b**)

A 55-year-old female patient visited a local clinic with a tick bite. She had no symptoms such as a headache, fever, nausea, or vomiting, but she had visited the local dermatology clinic on the day before our hospital visit to have the tick removed. She had been prescribed minocycline at the local clinic and had taken one dose in the evening before and one in the morning on the day of the visit to our hospital. She came to the Chosun University Hospital, Korea, outpatient clinic on June 19, 2018, for a second opinion. The patient was not sure of when she had been bitten by the tick. On the basis of her statement, that she had worked in fields 10–15 days before the hospital visit, we suspected that the tick had been on her for ~ 10 days. During the physical examination, we found a tick bite site on the lower part of her right clavicle. Although the tick was disposed of after it was removed, she brought a picture of the tick after the removal (Fig. 1).

Although we could not accurately classify the tick morphologically or genetically, it was highly likely a nymph of either *Amblyomma* spp. or *Haemaphysalis* spp., which are common in Korea. All the laboratory test results were within reference ranges: the first blood test results revealed a white blood cell (WBC) count of 5.1 × 10^3^/μL, hemoglobin of 13.8 g/dL, and a platelet count of 2.47 × 10^5^/μL; the blood biochemical test results showed aspartate aminotransferase (AST) at 17.9 U/L, alanine aminotransferase (ALT) at 17.1 U/L, γ-glutamyltransferase at 21 U/L, total bilirubin at 0.48 mg/dL, alkaline phosphatase (ALP) at 56 U/L, glucose of 86 mg/dL, blood urea nitrogen of 13.3 mg/dL, creatinine at 0.66 mg/dL, cholesterol at 211 mg/dL, and triglycerides at 98 mg/dL.

Although the patient was asymptomatic, we tested for tick-borne infectious diseases, e.g., anaplasmosis and rickettsiosis, by nested PCR (nPCR) and serological assays.

### nPCR

After extracting genomic DNA from the patient’s blood sample using the QIAamp Tissue and Blood Mini Kit (Qiagen, Hilden, Germany), nPCR was conducted using *Ehrlichia*- or *Anaplasma*-specific primers: the primer pairs GRO607F/GRO1294R and GRO677F/GRO1121R [10], which target the *groEL* (heat shock protein chaperone) gene; primer pairs ANK-F1/ANK-R1 and ANK-F2/ANK-R2 [11], which target the *ankA* (ankyrin-repeat protein) gene; and primer pairs AE1-F/AE1-R and AP-F/AP-R, which target the 16S ribosomal RNA (rRNA) gene [12].

To detect SFG rickettsiosis, nPCR was carried out using primer pairs sca1-6545F/sca1-7360R and sca1-6647F/sca1-7354R, which target the *sca1* (rickettsial surface protein) gene, and primers RR190.70F, RR190.602R, and RR190.701R [13], which are specific to the *ompA* gene. All primers that target the *sca1* gene were designed after sequence alignment to amplify this genomic region of all *Rickettsia* spp. The PCR products were separated by electrophoresis on a 1.2% agarose gel. In each PCR run, the reaction mixture without the template DNA served as a negative control. The genomic DNAs of *A. phagocytophilum* KZ_A3 and *Rickettsia conorii* were employed as positive controls for *Anaplasma*-specific and SFG *Rickettsia*–specific targets, respectively. The details of the experimental conditions are presented in Table 1.

**Table 1:** Nested PCR (nPCR) conditions as well as oligonucleotide primers used in this study

Target gene^**a**^	Primer name (sequences; 5′ → 3′)	Amplicon size (bp)
*groEL* nPCR for Anaplasmataceae (external primers)	GRO607F (GAAGATGCWGTWGGWTGTACKGC) GRO1294R (AGMGCTTCWCCTTCWACRTCYTC)	539
*groEL* nPCR for Anaplasmataceae (internal primers)	GRO677F (ATTACTCAGAGTGCTTCTCARTG) GRO1121R (TGCATACCRTCAGTYTTTTCAAC)	450
16S rRNA nPCR for *Anaplasma* and *Ehrlichia* species (external primers)	AE1-F (AAGCTTAACACATGCAAGTCGAA) AE1-R (AGTCACTGA CCCAACCTTAAATG)	1406
16S rRNA nPCR for *A*. *phagocytophilum* (internal primers)	AP-F (GTCGAACGGATTATTCTTTATAGCTTGC) AP-R (CCCTTCCGTTAAGAAGGATCTAATCTCC)	926
*ankA* nPCR for *A*. *phagocytophilum* (external primers)	ANK-F1 (GAAGAAATTACAACTCCTGAAG) ANK-R1 (CAGCCAGATGCAGTAACGTG)	705
*ankA* nPCR for *A*. *phagocytophilum* (internal primers)	ANK-F2 (TTGACCGCTGAAGCACTAAC) ANK-R2 (ACCATTTGCTTCTTGAGGAG)	664
*sca1* nPCR for SFG *Rickettsia* (external primers)	sca1-6545F (ATTCGTAACAGATTAGATRC) sca1-7360R (TTATAGGATGTTTTCGGTTG)	815
*sca1* nPCR for SFG *Rickettsia* (internal primers)	sca1-6647F (TGGATGCGTGSTATGTACG) sca1-7354R (GATGTTTTCGGTTGYTTCGG)	707
*ompA* nPCR for SFG *Rickettsia* (external primers)	R190.70F (ATGGCGAATATTTCTCCAAAAA) RR190.701R (GTTCCGTTAATGGCAGCATCT)	634
*ompA* nPCR for SFG *Rickettsia* (internal primers)	R190.70F (ATGGCGAATATTTCTCCAAAAA) RR190.602R (AGTGCAGCATTCGCTCCCCCT)	535

^a^*ankA* ankyrin-repeat protein gene, *groEL* heat shock protein chaperone gene, *rRNA* ribosomal RNA, *sca1* surface cell antigen 1 (rickettsial surface protein) gene, *ompA* outer membrane protein A gene

### Serological testing

An indirect immunofluorescence assay (IFA) was performed for the serological diagnosis of the patient. To detect antibodies to SFG *Rickettsia*, we utilized antigen slides of *R*. *conorii*, *R. japonica*, and *R*. *montanensis*. To perform the IFA, 20 μL of two-fold serial dilutions from 1:16 of the patient’s serum was reacted with each rickettsial antigen slide in a humidified chamber at 37 °C for 30 min. The antigen slides were washed three times with PBS and then another three times with distilled water and were air-dried. Next, the slides were incubated with 20 μL of a 400-fold–diluted secondary antibody (a fluorescein isothiocyanate [FITC]-conjugated anti-human IgG or IgM antibody), washed, and air-dried in the same manner as mentioned above. The slides fixed with a mounting medium were visualized under a fluorescence microscope (U-LH100HG, Olympus Corp., Tokyo, Japan) to detect SFG *Rickettsia*-specific fluorescence.

To diagnose anaplasmosis, an IFA was performed in a similar manner on an antigen slide containing an *A*. *phagocytophilum* strain. A four-fold or greater increase in the antibody titer in the acute-phase and convalescent-phase serum samples was assumed to be a positive indicator of SFG rickettsiosis and anaplasmosis [1].

**Fig. 2:**
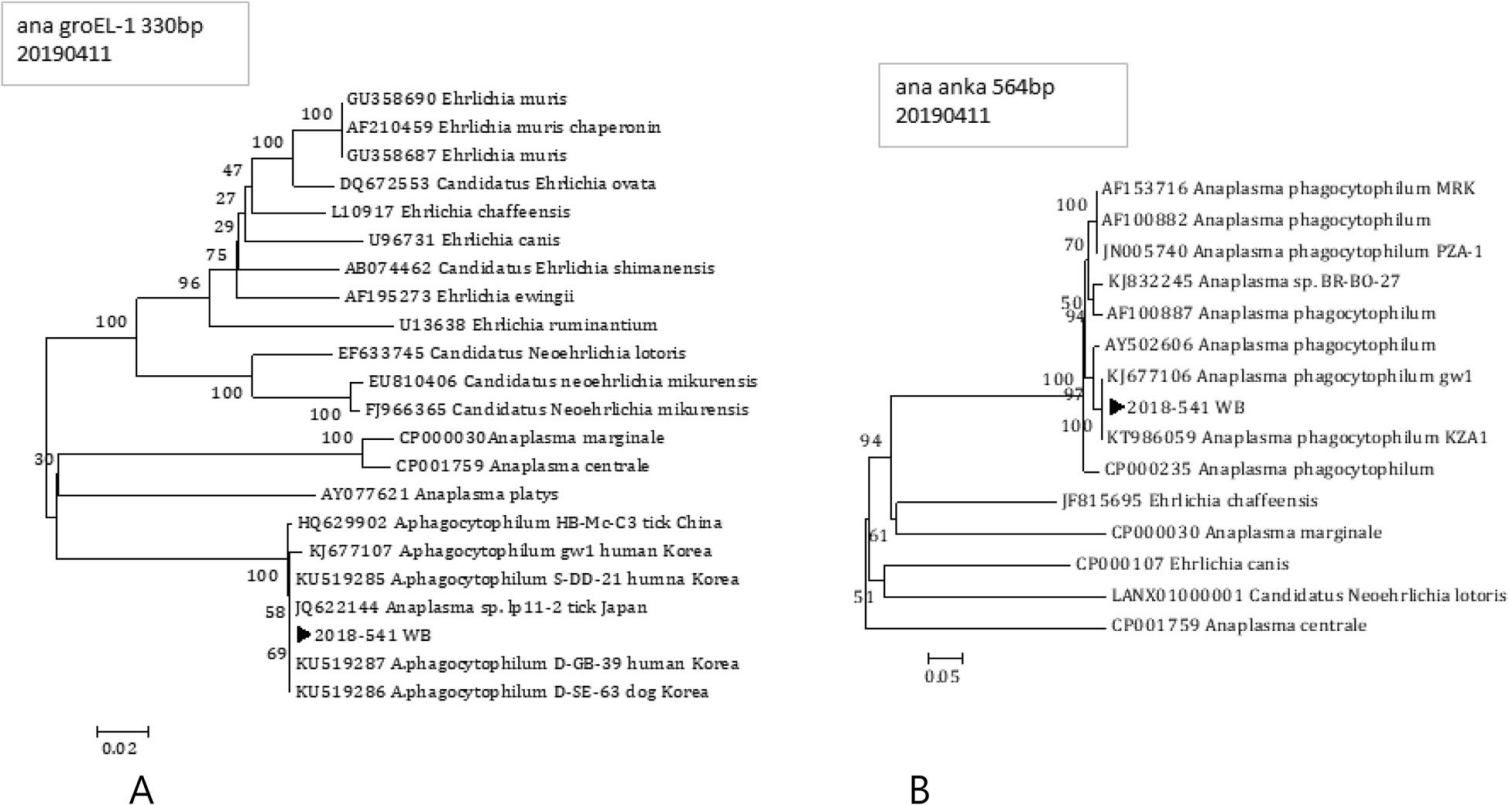
Phylogenetic-tree positions of the *A*. *phagocytophilum*–specific *groEL* and *ankA* sequences in the patient. The arrowhead indicates our strain

The nPCR that was performed on the patient’s first visit (June 19) yielded a positive result on the *Ehrlichia*- or *Anaplasma*-specific *groEL* and *ankA* genes; however, the nPCR targeting the 16S rRNA gene gave a negative result. DNA sequencing of the positive-result PCR products from the patient showed that the *groEL* gene sequence was a 100% match (332 out of 332 bp) for *A. phagocytophilum* isolates “S-DD-21,” “D-SE-63,” “D-GB-39,” and “lp11–2” (GenBank accession numbers KU519285, KU519286, KU519287, and JQ622144, respectively). Genotype S-DD-21, D-SE-63, and D-GB-39 were originally identified in Korean cats and dogs, and isolate lp11–2 originates from a Japanese tick. Isolate gw1, which was originally collected from a Korean patient, had the second-highest homology with our strain, and phylogenetic-tree analysis showed that our strain belongs to the same group as *A. phagocytophilum* (Fig. 2a). The *ankA* gene sequence from the microbe(s) found in our patient was 99.8% (558/559 bp) homologous to that of *A. phagocytophilum* isolates gw1 and KZA1 (accession numbers KJ677106 and KT986059, respectively), which were originally collected from a Korean patient. Moreover, the phylogenetic-tree analysis confirmed that the *ankA* sequence places our microbial isolate into the same group as *A. phagocytophilum* (Fig. 2b). DNA sequence analysis and the phylogenetic tree based on the *groEL* and *ankA* sequences confirmed the presence of *A*. *phagocytophilum* in the patient, despite the lack of symptoms. By contrast, in the PCR assay, the same blood sample collected during the patient’s hospital visit tested negative for *Rickettsia*-specific *ompA* and *sca1*.

To isolate the bacterial strain, we performed an animal experiment. The patient’s blood was intraperitoneally injected into a BALB/c mouse obtain from ORIENT BIO (Seongnam-si, Gyeonggi-do, Korea), treated with cyclophosphamide (Sigma-Aldrich, St. Louis, MO, USA). The mouse was euthanized by inhalation of 5% isoflurane and then dissected, subsequent nPCR performed on the mouse’s spleen, kidney, and liver yielded a negative result on the *Rickettsia*-specific *sca1* gene and *Anaplasma*-specific genes *groEL* and *ankA*.

The IFA for antibodies to an *A*. *phagocytophilum* antigen on June 19th (first visit) gave a negative result on both IgG and IgM (Table 2). By contrast, the IFA for an anti-*A*. *phagocytophilum* antibody performed on June 22nd (second visit) yielded an IgG-negative but IgM-positive result (1:16). On June 28th (third visit), seroconversion was confirmed because the serum sample was IgG negative and IgM positive (1:32). The anti-*R*. *conorii* antibody IFA performed on June 28th showed an elevated (to 1:64) IgG titer [14].

**Table 2:** Nested PCR (nPCR) and indirect immunofluorescence assay (IFA) results for anaplasmosis and rickettsiosis

Date	Anaplasmosis			Rickettsiosis						
	nPCR	IFA		nPCR	IFA (***R***. ***conorii***)		IFA (***R. japonica***)		IFA (***R***. ***montanensis***)	
		IgG	IgM		IgG	IgM	IgG	IgM	IgG	IgM
**19 June**	*groEL* (+) *ankA* (+)16S rRNA (−)	< 1:16	< 1:16	*sca1* (−) *ompA* (−)	< 1:16	**1:256**	**1:128**	< 1:16	**1:32**	< 1:16
**22 June**		< 1:16	**1:16**	*sca1* (−)	< 1:16	**1:512**	**1:128**	< 1:16	**1:32**	< 1:16
**28 June**	*groEL* (−) *ankA* (−)	< 1:16	**1:32**	*sca1* (−)	**1:64**	**1:256**	**1:64**	< 1:16	**1:32**	< 1:16

*nPCR* nested PCR, *IFA* indirect immunofluorescence assay

## Discussion and conclusion

Ticks in the Ixodidae family can serve as vectors of *A*. *phagocytophilum* and SFG *Rickettsia*. They usually live in tropical regions as well as in South Asia, Japan, and Korea. In Korea, they are known to be found, for example, on Jeju Island, in Suncheon (South Jeolla Province), Tongyoung, and Changwon (Gyeongsang Province). The first case of a tick bite in Korea was reported in Damyang-gun, South Jeolla Province [15]. Previous reports indicate that larvae require 3 to 5 days to have a meal of their hosts’ blood. After the metamorphosis of the larvae, a nymph requires 7 to 10 days to transform into an imago; before shedding, an imago requires feeding on its respective host’s blood for 6 to 13 days. Unlike *Borrelia burgdorferi*, *A. phagocytophilum* and SFG *Rickettsia* get inoculated into a host within 2 to 6 h of attachment following a tick bite [16].

The serological diagnosis of an *A*. *phagocytophilum* infection can be confirmed when there is a four-fold increase in the antibody titer in convalescent-phase serum compared to that in acute-phase serum [17, 18]. Because the increase in the plasma concentration of antibodies in response to *A*. *phagocytophilum* proceeds slowly, a significant increase is not detectable in the acute phase. Furthermore, there are cases where rescreening for antibodies should be performed at ~ 1 month after the original test [18, 19]. In the anaplasmosis IFA, our patient tested negative on the first visit, but IgM seroconversion was confirmed in the follow-up tests on June 22nd and June 28th. Moreover, although the patient presented no symptoms, the nPCR results were positive for the *A*. *phagocytophilum*–specific genes *groEL* and *ankA*; accordingly, the patient received a diagnosis of asymptomatic *A*. *phagocytophilum* infection.

The anti-*R*. *conorii* antibody IFA performed on June 19th gave a negative result on IgG, but the IgG titer was found to have increased to a ratio of 1:64 in the IFA that was performed later, i.e., on June 28th. The IFA for antibodies to *R. japonica* and *R*. *montanensis* yielded positive results with low IgG titers. The *sca1* nPCR gave a negative result on *Rickettsia* on June 19. Nonetheless, because the patient had taken two doses of minocycline (a tetracycline antibiotic) since the tick bite, it was unclear whether SFG rickettsiosis could not be detected by PCR owing to the antibiotic. Therefore, we could not rule out the possibility of SFG *Rickettsia* coinfection and the possibility of a cross-reaction due to the *A*. *phagocytophilum* infection [19].

Currently, there are published cases of asymptomatic anaplasmosis in various animals, including camels, horses, dogs, and sheep [19]. In contrast, there are few studies on the genetic and serological diagnosis of this infection in humans. In a study involving 148 blood samples from people with HIV infection, Welc-Falęciak et al. confirmed two cases of asymptomatic infection by tick-borne pathogens: *Borrelia garinii* and *A*. *phagocytophilum* [9]. There is a report of asymptomatic anaplasmosis in people with HIV infection, but no study has shown a genetic and serological diagnosis of asymptomatic anaplasmosis in non-HIV patients.

One limitation of this study is that the asymptomatic infection could not be definitively diagnosed by the culture method. We attempted to culture *Anaplasma* and *Rickettsia* by means of a mouse but failed. Additional studies in healthy adults are needed to determine whether the failure of culturing was because of minocycline administration or the low *Anaplasma* cell numbers in an asymptomatic infection. Seroconversion of SFG rickettsiosis was confirmed in our patient, and further studies are needed to examine whether the antibodies to SFG *Rickettsia* that were produced in the acute phase of anaplasmosis were a result of a cross-reaction. Furthermore, additional studies are necessary to investigate whether the absence of symptoms in our patient with anaplasmosis was a consequence of taking minocycline during the latency period.

In conclusion, in this study, we diagnosed an asymptomatic *A*. *phagocytophilum* infection both genetically and serologically. Furthermore, although SFG rickettsiosis was not confirmed genetically, seroconversion was confirmed, suggesting a possible coinfection with SFG *Rickettsia*. Therefore, clinicians should be aware of the possibility of asymptomatic anaplasmosis after a tick bite.

## Data Availability

The datasets analysed during the current study are available at National Center for Biotechnology Information (NCBI) repository, (accession numbers; KU519285, KU519286, KU519287, JQ622144, KJ677106 and KT986059).

## Abbreviations

ALT: Alanine aminotransferase
ALP: Alkaline phosphatase
AnkA: Ankyrin-repeat protein
AST: Aspartate aminotransferase
GroEL: Heat shock protein chaperone
IFA: Indirect immunofluorescence assay
nPCR: Nested PCR
OmpA: Outer membrane protein A
Sca1: Rickettsial surface protein, surface cell antigen 1
SFG: Spotted fever group
WBC: White blood cell
